# Impact of habitat fragmentation on tsetse populations and trypanosomosis risk in Eastern Zambia

**DOI:** 10.1186/s13071-015-1018-8

**Published:** 2015-08-04

**Authors:** Cornelius Mweempwa, Tanguy Marcotty, Claudia De Pus, Barend Louis Penzhorn, Ahmadou Hamady Dicko, Jérémy Bouyer, Reginald De Deken

**Affiliations:** Department of Veterinary and Livestock Development, Zambia, Africa; Animal Health Department, Institute of Tropical Medicine, 2000 Antwerp, Belgium; Department of Veterinary Tropical Diseases, Faculty of Veterinary Science, University of Pretoria, Pretoria, South Africa; West African Science Service in Climate Change and Adapted Land Use (WASCAL), Climate change economics research program, Cheikh Anta Diop University, BP 5683 Dakar, Senegal; Centre de Coopération Internationale en Recherche Agronomique pour le Développement, Unité Mixte de Recherche Contrôle des Maladies Animales Exotiques et Emergentes, Campus International de Baillarguet, 34398 Montpellier, France; Institut National de la Recherche Agronomique (INRA), Unité Mixte de Recherche 1309 ‘Contrôle des Maladies Animales Exotiques et Emergentes’, 34398 Montpellier, France; Centre de Coopération Internationale en Recherche Agronomique pour le Développement (CIRAD), Unité Mixte de Recherche ‘Interactions hôtes-vecteurs-parasites-environnement dans les maladies tropicales négligées dues aux trypanosomatides’, 34398 Montpellier, France; Institut Sénégalais de Recherches Agricoles, Laboratoire National d’Elevage et de Recherches Vétérinaires, Service de Parasitologie, BP 2057 Dakar - Hann, Sénégal; VERDI-R&D, 4141 Louveigné, Belgium

**Keywords:** Habitat fragmentation, Tsetse ecology, Trypanosomosis risk, Zambia

## Abstract

**Background:**

Fragmentation of tsetse habitat in eastern Zambia is largely due to encroachments by subsistence farmers into new areas in search of new agricultural land. The impact of habitat fragmentation on tsetse populations is not clearly understood. This study was aimed at establishing the impact of habitat fragmentation on physiological and demographic parameters of tsetse flies in order to enhance the understanding of the relationship between fragmentation and African animal trypanosomosis (AAT) risk.

**Methods:**

A longitudinal study was conducted to establish the age structure, abundance, proportion of females and trypanosome infection rate of Glossina morsitans morsitans Westwood (Diptera: Glossinidae) in areas of varying degrees of habitat fragmentation in Eastern Zambia. Black screen fly rounds were used to sample tsetse populations monthly for 1 year. Logistic regression was used to analyse age, proportion of females and infection rate data.

**Results:**

Flies got significantly older as fragmentation increased (*p* < 0.004). The proportion of old flies, i.e. above ovarian category four, increased significantly (*P* < 0.001) from 25.9 % (CI 21.4–31.1) at the least fragmented site (Lusandwa) to 74.2 % (CI 56.8–86.3) at the highly fragmented site (Chisulo). In the most fragmented area (Kasamanda), tsetse flies had almost disappeared. In the highly fragmented area a significantly higher trypanosome infection rate in tsetse (*P* < 0.001) than in areas with lower fragmentation was observed. Consequently a comparatively high trypanosomosis incidence rate in livestock was observed there despite lower tsetse density (*p* < 0.001). The overall proportion of captured female flies increased significantly (*P* < 0.005) as fragmentation reduced. The proportion increased from 0.135 (CI 0.10–0.18) to 0.285 (CI 0.26–0.31) at the highly and least fragmented sites, respectively.

**Conclusions:**

Habitat fragmentation creates conditions to which tsetse populations respond physiologically and demographically thereby affecting tsetse-trypanosome interactions and hence influencing trypanosomosis risk. Temperature rise due to fragmentation coupled with dominance of old flies in populations increases infection rate in tsetse and hence creates high risk of trypanosomosis in fragmented areas. Possibilities of how correlations between biological characteristics of populations and the degree of fragmentation can be used to structure populations based on their well-being, using integrated GIS and remote sensing techniques are discussed.

## Background

Ecological and environmental factors such as host availability, predation, shelter, temperature and humidity are critical in determining the dynamics of a tsetse population [1, 2]. These factors are affected by the degree of habitat fragmentation [3, 4]. Habitat fragmentation is here defined as the breaking up of habitat, particularly native vegetation, into smaller isolated fragments. Habitat fragmentation on the plateau of eastern Zambia is driven mainly by human encroachment into bushland in search of land for livestock breeding and crop production. Changes in the environment have repercussions on the distribution and density of tsetse [5] and possibly also on other demographic parameters of tsetse such as sex ratio, age structure and body size. Demographic status may reflect the stress level of a population. For example, having a higher proportion of young than old flies in a tsetse population is an indicator of a population with a high renewal rate. Age structure and body size of teneral flies are some of the parameters that reflect a population’s response to various ecological conditions [1, 6, 7]. Other notable responses of tsetse to ecological conditions include (i) increase in the proportion of female flies captured by fly round methods in a population as a result of nutritional stress [8]; (ii) life expectancy being longer in cool than in hot seasons [9]; (iii) body size of the offspring (as measured by wing vein length) that may change depending on the conditions experienced by the parent flies during the period preceding the capture [10, 11].

Previously, it was demonstrated that fragmentation intensity negatively impacts tsetse apparent densities [12]. A similar impact was also observed in riverine species of tsetse in West Africa [13].

Hence, by comparing parameters representing the degree of habitat fragmentation with parameters representing the status of the tsetse population (e.g., age structure, body size, abundance, sex ratio, etc.) and their infection rate, the possible impact of fragmentation on these factors may be established. Hence repercussions of such relationships on the epidemiology of trypanosomosis can possibly be applied to the planning of control operations.

In this paper, the impact of habitat fragmentation on the status of age structure, tsetse abundance, proportion of female flies and infection rate is investigated in order to study the relationship between fragmentation and AAT risk.

## Methods

### Study area

**Fig. 1:**
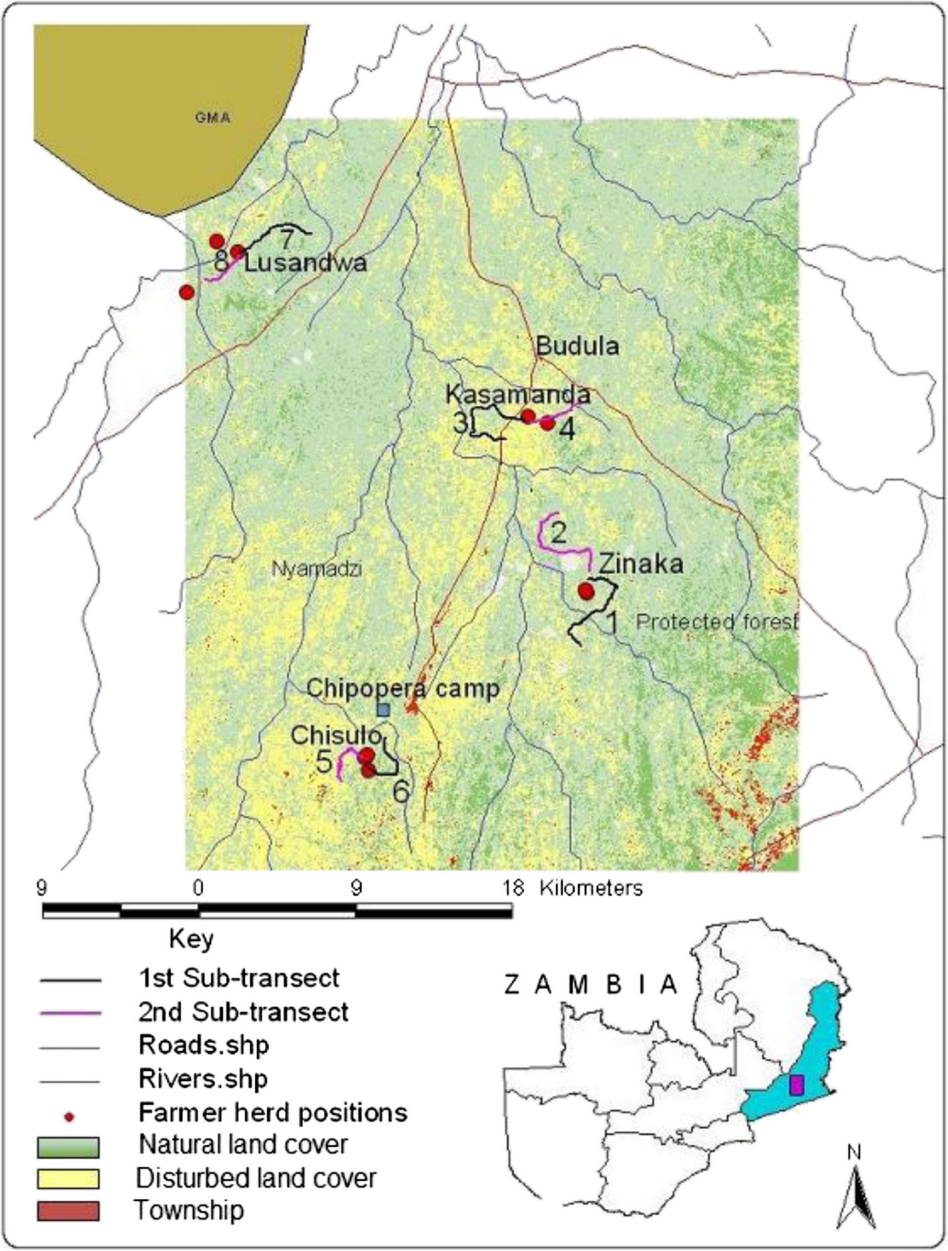
Location of the study area

The study was carried out from July 2006 to November 2007 in an area located between 31°47’–31°55’ E and 13°55’–14°12’ S with an altitude between 750 and 1000 m in two districts (Katete and Mambwe) of the eastern province of Zambia (Fig. 1). Visually in the field, a reducing gradient of habitat loss [due to human encroachment] was evident from the south to the north.

Two tsetse species, *Glossina morsitans morsitans* and *G. pallidipes*, are prevalent in the study area with *G. pallidipes* occurring in high densities only in the north [14]. Three climatic seasons can be distinguished: a warm rainy season from late November to April, a cold dry season from May to August and a hot dry season from September to October/early November.

In the study area, the cattle density decreases from about 8.2/km^2^ in the south to about 2.8/km^2^ in the north (based on a livestock census conducted in 2006).

### Fragmentation and selection of longitudinal study sites

Based on a fragmentation classification and the distribution of tsetse in the study area [12], four sites, representing an increasing gradient of habitat change, were selected: Lusandwa, Zinaka, Chisulo and Kasamanda. Ducheyne et al. [12] reported the majority of tsetse flies being caught from the less fragmented area and catches reducing with fragmentation. To characterize the type and extent of fragmentation at specific study sites, the landscape characteristics, which included relative cover area, number of patches of disturbed or undisturbed areas per km^2^ and mean patch size, were determined using the methods described by Ducheyne et al. [12], at three different radii (0.5 km, 1.3 and 4.8 km) from the centre of each the study site. The 4.8 km radius encompassed the two sub-transects along which tsetse flies were sampled (see section 2.3 below) and the 0.5 km is a daily root mean square random displacement of *Glossina morsitans morsitans* [15]. The 1.3 km was an arbitrary distance.

### Tsetse population monitoring

At each of the four study sites, sampling of tsetse flies was carried out by black screen fly rounds (traverses) along two sub-transects (1 to 8) of a total length ranging from 6 to 11.5 km that meandered through various ecotones (Fig. 1). Transects were traceable by paint marks on trees and stones. Each sub-transect was divided into sectors of 110–120 m in length. The start and end of each sector was marked, numbered and geo-referenced. The two sub-transects at each site were traversed by the same pair of men who caught tsetse flies attracted to the black screen [16] with the difference that in this study the screen was baited with attractants butanone [17] and octenol [18]. Transects were traversed at an average of 8 times per month on alternating days and times of the day (morning or afternoon) during the last half of each month. From tsetse catches at stops an estimate of relative size of tsetse population [index of apparent abundance (IAA)] was calculated as the numbers caught per stop in each traverse.

Flies caught were examined for trypanosomal infection by examination of mouth parts, mid-guts and salivary glands for trypanosomes [19] using compound microscopes. Flies found infected with trypanosomes in the midgut only, were considered to have an immature infection.

### Age estimation

Individual female flies were allocated to one of the 16 ovarian categories (0–15) depending on the configuration of the ovary and the content of the uterus (egg, 1st instar larva, 2nd instar larva or 3rd instar larva) [20]. Flies of ovarian category 4 and above were considered old.

### Trypanosomosis monitoring

A sentinel herd of 40 cattle (Fig. 1) was established at each of the four longitudinal study sites to monitor trypanosomosis incidence. At setting up of sentinel herds all animals were ear-tagged and treated with diminazene aceturate (Berenil®, Hoechst) at 7 mg/kg body weight. Examination for trypanosomosis was carried out at monthly intervals for a period of 12 months. Blood was collected from the lower ear-vein in two heparinised microhaematocrit capillary tubes which were then sealed at one end with Cristal seal. Blood was then centrifuged in a microhaematocrit centrifuge for 5 min at 9000 rpm. The packed cell volume (PCV) was read and the buffy coat wet smears were examined under bright field illumination to diagnose infection.

Animals found harbouring trypanosomes were treated on the spot with diminazene aceturate at a standard dose of 3.5 mg/kg body weight. Animals with PCV less than 22 were also treated with the same drug and dosage as they were considered likely to be infected.

### Data analysis

Of the two tsetse species found in the area, only *G. morsitans morsitans* data were subjected to analysis as *G. pallidipes* was not caught at all study sites. Further data from the Kasamanda study site were excluded from most analyses because of the very low number of flies caught.

Proportions of female flies of various ovarian categories were analysed using ordered multinomial logistic regression analysis while infection rate, sex proportions, apparent abundance and proportion of old female flies were analysed using logistic regression. Explanatory variables were season and sites in all models. Interactions between season and sites were taken into account.

Spatial and temporal changes in measured tsetse parameters/characteristics were checked for correlation with the degree of habitat fragmentation to test a possible effect of fragmentation.

The monthly trypanosomosis incidence was expressed as a proportion of newly infected animals excluding those treated with diminazene aceturate the previous month. Incidence data were analysed using logistic regression.

The relative risks and their confidence intervals represented by the product of the mean index of apparent abundance multiplied by the rate of infection of the flies [21] [i.e., the entomological inoculation rate (EIR)] were obtained from bootstrapping (1000 Monte Carlo simulation, R software) in the apparent abundance distributions and from fly dissection results at each site and season, assuming spatial homogeneity within a given epidemiological landscape. All confidence intervals were calculated for a risk α of 5 %.

To determine the association between trypanosomosis incidence rate in cattle and trypanosome infection status in tsetse populations, a multiple logistic regression analysis was used where the disease status of animals during examinations was the response variable and season and infection rate in tsetse populations at study sites were predictor variables. Adjusted Odds ratios (OR) were used to measure association.

## Results

### Habitat fragmentation

**Fig. 2:**
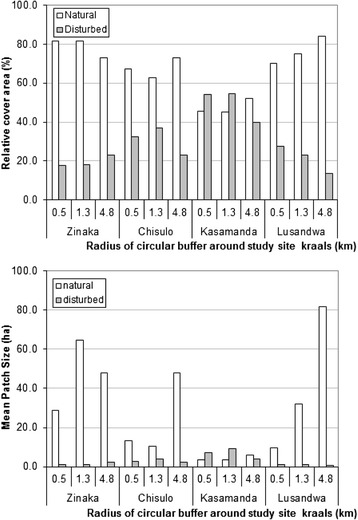
Relative cover area and mean patch size

**Fig. 3:**
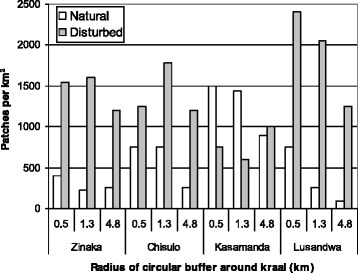
Natural and disturbed patch density

Vegetation cover within various radii showed that the Lusandwa and Zinaka study sites were marginally disturbed, as indicated by the vegetation relative cover area (Fig. 2). The disturbed cover increased farther away from the centre of the study site in Zinaka while it reduced farther away in Lusandwa. The disturbed relative cover area at Kasamanda exceeded the natural cover area within 1.3 km radius and was about 75 % of the natural cover farther away (4.8 km radius). At Chisulo, the disturbed relative cover area was about half of the natural relative cover area within 1.3 km radius and fell to about 20 % farther away (4.8 km radius). With regard to patch sizes, Zinaka study site had large natural patches in all circular radii but at Chisulo and especially in Lusandwa, large patches were found in the 4.8 km radius. Kasamanda study site had the highest number of natural patches per km^2^ (Fig. 3), but these patches were small (Fig. 2). Natural patches at Chisulo were larger and thus scarcer than at Kasamanda. Hence Kasamanda area was the most fragmented, followed by Chisulo area, then Zinaka area and Lusandwa was the least fragmented area, especially at its outskirts. Lusandwa (a) was also the closest to the Luangwa National Park game management area (GMA) (Fig. 1).

For ease of remembering variations in overall degree of habitat fragmentation, a letter a to d in brackets is assigned to each study site name in increasing order of fragmentation as follows: Lusandwa (a), Zinaka (b), Chisulo (c) and Kasamanda (d).

### Index of apparent abundance

Transects used to sample tsetse flies had a total of 348 numbered stops [64, 116, 74 and 94 at Lusandwa (a), Zinaka(b), Chisulo (c) and Kasamanda (d)], respectively. A total of 275.75 fly rounds (traverses) were carried out [85.75, 95, 95, and 84.24 at Lusandwa (a), Zinaka (b), Chisulo (c) and Kasamanda (d), respectively]; due to rain disturbances some fly rounds were not completed, hence the decimal numbers. A total of 3200 *Glossina m. morsitans* were caught. Respective numbers caught were 1835 at Lusandwa (a), 1104 at Zinaka (b), 252 at Chisulo (c) and 9 at Kasamanda (d).

**Fig. 4:**
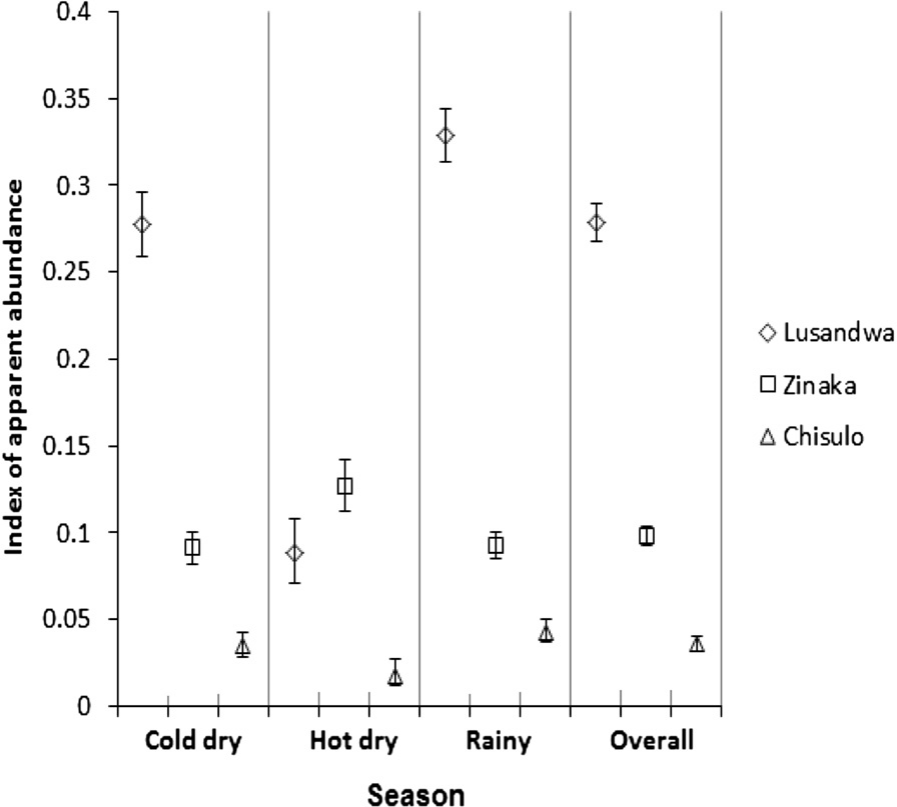
Index of apparent abundance

The overall IAA increased as fragmentation decreased, i.e. 0.001, 0.0357, 0.0977 and 0.2784 at Kasamanda (d), Chisulo (c), Zinaka (b) and Lusandwa (a), respectively (Fig. 4). The same order of IAA (from lowest to highest) was observed in all seasons. The IAA dropped by 4 times in Lusandwa (a) and increased by 1.4 times in Zinaka (b) during the hot dry season from levels in the rainy season. Significant differences in the IAA were observed between Lusandwa (a) samples and those of the three sites (*P* < 0.001).

### Ovarian age structure of tsetse populations

**Fig. 5:**
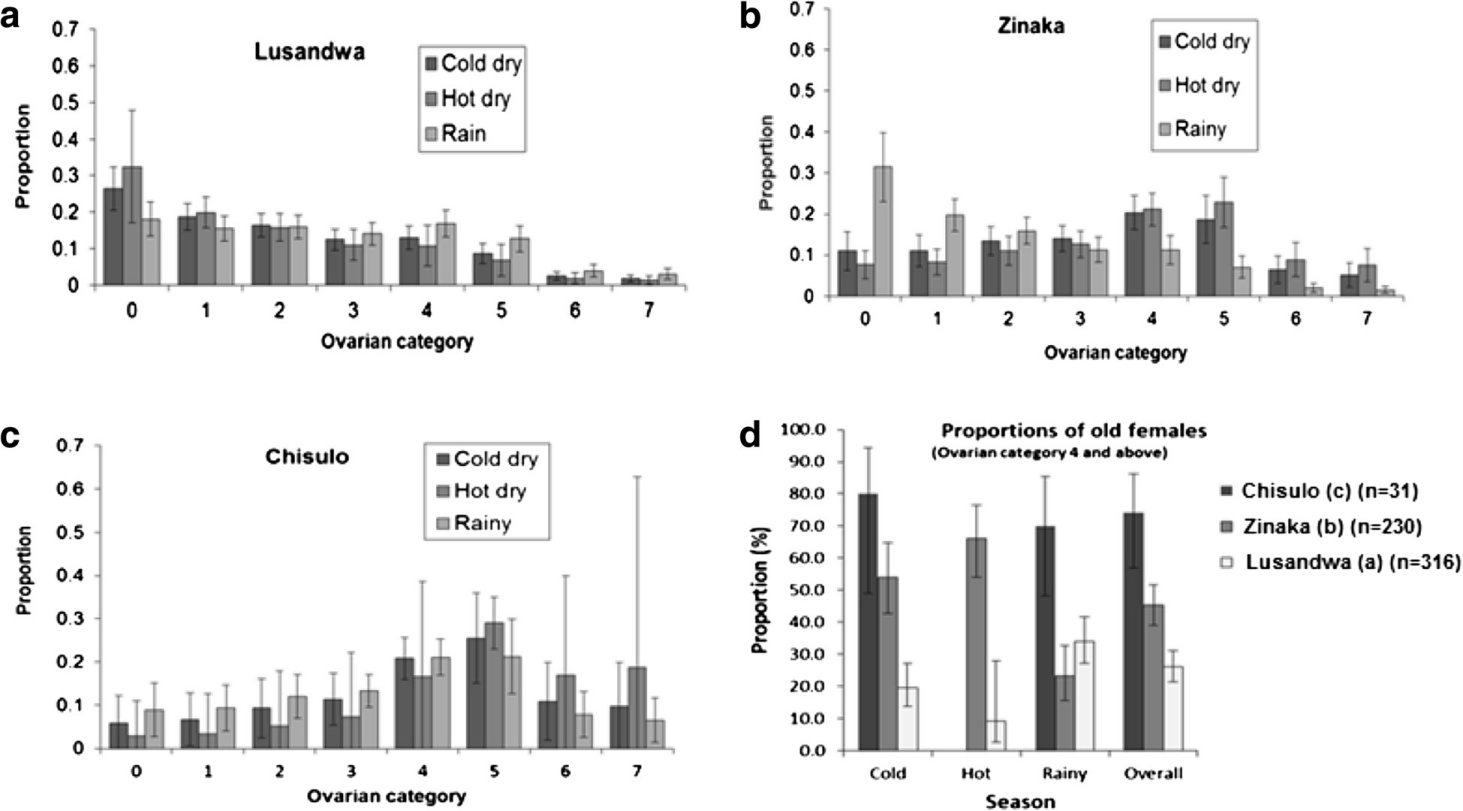
Seasonal proportions of female flies of various ovarian categories derived from the model. **a** Depicts right skew of proportions in all seasons; **b** Depicts right skew of proportions in the rainy season and left skew in the cold and hot dry season; **c** Depicts left skew of proportions in all seasons and **d** Depicts the similarity in pattern of increase between the proportion of old flies and degree of habitatfragmentation.

A total of 577 female *G. m. morsitans* were dissected and their ovarian age determined: 31, 316 and 230 in Chisulo (c), Lusandwa (a) and Zinaka (b), respectively. The ordered multinomial logistic regression of ovarian category on site and season produced age distributions of female flies that varied considerably between study sites. In Lusandwa (a) the age structure was skewed to the right in all seasons (Fig. 5a). In Chisulo (c), the age distribution was the opposite of that in Lusandwa (a) (skewed to the left in all seasons) (Fig. 5c). The Zinaka (b) population showed a combination of Lusandwa (a) and Chisulo (c) population structures. The age distribution resembled that of Lusandwa (a) population only in the rainy season and that of Chisulo (c) population in the other two seasons (Fig. 5b). The pattern of increase in overall proportion of old flies (ovarian category four and above—a section of the population with usually a high infection rate [22, 23] correlated positively with the pattern of increase in degree of habitat fragmentation (Fig. 5d). The status could not be ascertained in the hot dry season at Chisulo (c) as only one female fly was dissected for ageing. Proportions of old flies (ovarian category four and above) were significantly different between sites (*p* < 0.001) with the population at Lusandwa (a) having the lowest [25.9 % (CI 21.4–31.1)] and that at Chisulo (c) the highest [74.2 % (CI 56.8–86.3)]. The proportion at Zinaka (b) was 45.2 % (CI 38.9–51.7). The overall mean age of female flies was 39.7 ± 3.4 days for Chisulo (c) sample (*n* = 31), 32.0 ± 1.5 days for Zinaka (b) (*n* = 230) and 26.1 ± 1.0 days (*n* = 317) for Lusandwa (a) samples. The population was older in Chisulo (c) than in Zinaka (b) and that in Zinaka (b) was older than that in Lusandwa (a) (Fig. 5d) (*p* < 0.004).

### Proportion of female flies

**Fig. 6:**
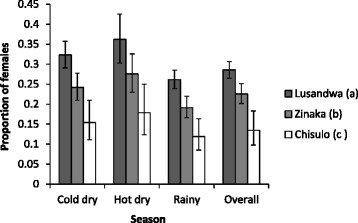
Proportion of female flies at study sites and in different seasons

Excluding Kasamanda (d) data, a total of 3191 flies were used for determination of the proportion of female flies: 807 females (524, 249 and 34 at Lusandwa (a), Zinaka (b) and Chisulo (c), respectively) and 2384 males (1311, 855 and 218 at Lusandwa (a), Zinaka (b) and Chisulo (c), respectively). The proportion of captured female flies correlated inversely with the degree of fragmentation from 0.135 (CI 0.10–0.18) at Chisulo (c) to 0.285 (CI 0.26–0.31) at Lusandwa (a) and this inverse correlation was observed in all seasons (Fig. 6). Overall, the variation in proportion of female flies between all pairs of site populations were significant (*P* < 0.005). In each season, the variation in female proportion between the most and least fragmented site was significant (*P* < 0.001).

### Infections by trypanosomes

#### Tsetse flies

A total of 2215 flies were dissected for trypanosome infection examination. Among these, 924 were examined at Lusandwa (a), 1064 at Zinaka (b), and 227 at Chisulo (c). A total of 153 flies (70, 51 and 32 at Lusandwa (a), Zinaka (b) and Chisulo (c), respectively) and 261 flies (207, 23 and 31 at Lusandwa (a), Zinaka (b) and Chisulo (c), respectively) were found with mature and immature infections, respectively. Overall, 28.1 % (95 % CI 22.6–34.2 %), 7.0 % (5.6–8.6) and 30.0 % (27.1–33.0) of *G. m. morsitans* in Chisulo (3), Zinaka (2) and Lusandwa (1), respectively, were found with trypanosomes. Infections of the mid-gut only were the most common at all study sites. The number of flies with infections in the mid-gut only (immature infections) were significantly high at Lusandwa (a) 207 (22.4 % (CI 19.8–25.2) and Chisulo (c) 31 (13.7 % CI 9.8–18.7) compared to the number at Zinaka (b) 23 (2.2 % CI 1.4–3.2) (*P* < 0.001). The second most common organ infections were those of the mid-gut + mouth part at Zinaka (b) 36 (3.4 % CI 2.5–4.6) and Chisulo (c) 18 (7.9 % CI 5.1–12.2) while at Lusandwa (a) it was the salivary gland only and mouth part only with 26 and 25 flies (2.9 % CI 2.0–4.2 and 2.7 % CI 1.8–4.0), respectively.

The classical dissection technique used [20] showed that the prevalence, in percentage, of infections typical of *T. congolense* group were significantly different between sites (*P* = 0.002) with the highest at the highly fragmented site Chisulo (c) (7.9 % (CI 5.1–12.1)) followed by that at Zinaka (b) (3.4 % ((CI 2.5–4.6)) and lowest at the least fragmented site Lusandwa (a) (0.6 % (CI 0.2–1.4)). The prevalence of infections typical of *T. vivax* were significantly different between Lusandwa (a) and Zinaka (b) samples (*P* = 0.001) with 2.7 % (CI 1.8–4.0) and 0.9 % (CI 0.5–1.7), respectively [Chisulo (c) samples had 0.0 % infection typical of *T. vivax*]. The prevalence of infections typical of *T. brucei* group (including flies infected in all body parts) at Lusandwa (a) 4.3 % (CI 3.2–5.8) and Chisulo (c) 6.2 % (CI 3.7–10.1) were significantly higher than those at Zinaka (b) 0.5 % (CI 0.2–1.1) (P < 0.001).

**Fig. 7:**
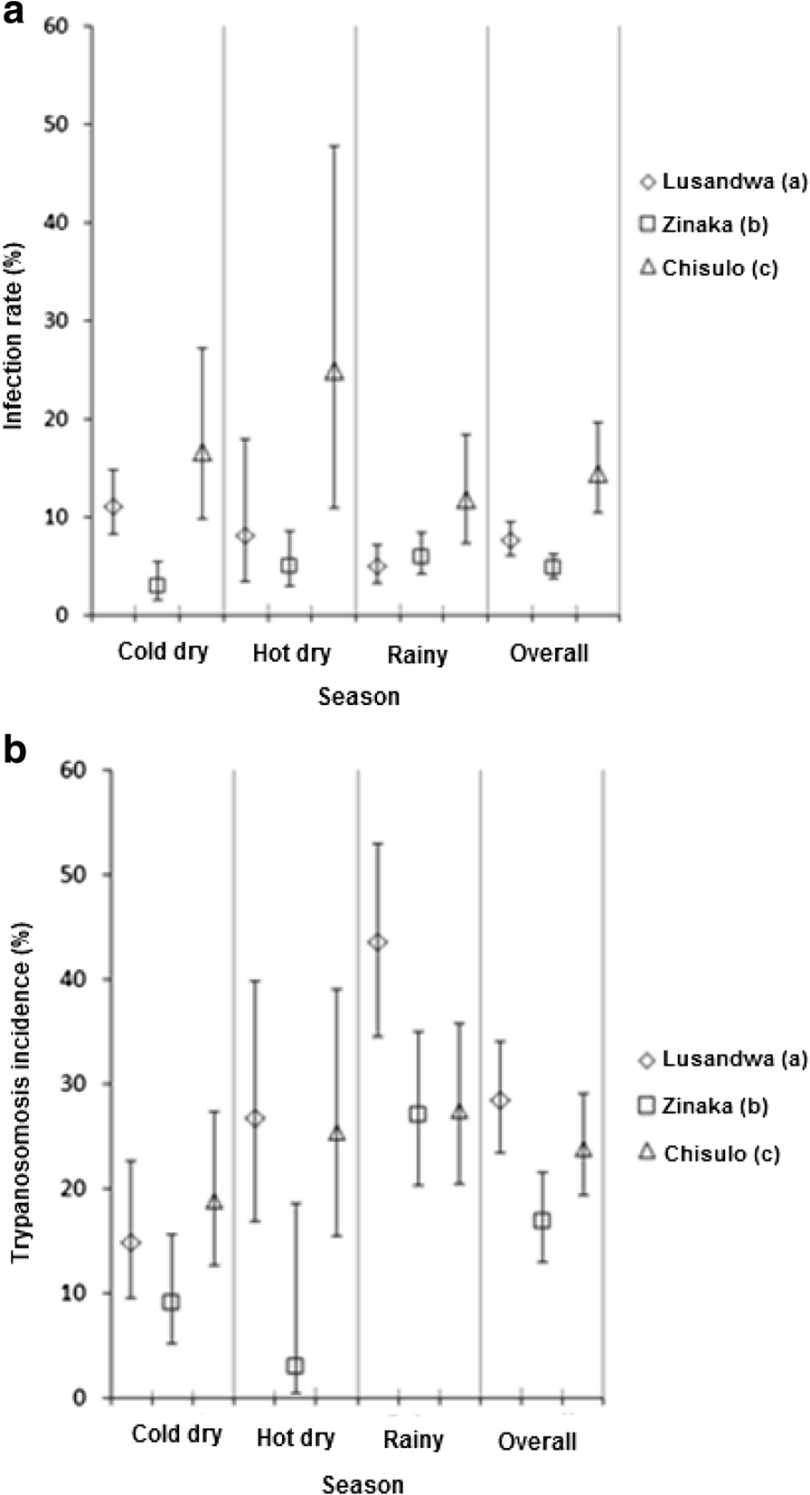
Infection rates by trypanosomes. (**a**) Tsetse (**b**) Cattle

The mature infection rate (excluding immature infections) was highest at the highly fragmented site [Chisulo (c)] at 14.5 % (CI 10.5–19.6), followed by the least fragmented site [Lusandwa (a)] at 7.6 % (CI 6.0–9.5) and lowest at the intermediately fragmented site [Zinaka (b)] at 4.8 % (CI 3.7–6.2). No correlation could be observed between mature infection rate and the degree of habitat fragmentation for the different sites and for the sites within seasons (Fig. 7a). Significant differences in mature infection rate were observed between Chisulo (c) and the other two sites (*P* = 0.001). Further, in each season the infection rate in Chisulo (c) was significantly higher than that in Zinaka (b) (*P* < 0.001).

#### Cattle

A total of 277 positive cases (infected animals) were diagnosed on buffy coat examination. Of these cases, 38 were animals treated with Berenil® at 3.5 mg/kg body weight the previous month for being infected or having low PCV. All infections were due to *T. congolense* except for one due to *T. vivax* in Lusandwa (a) and three mixed infections of Tv/Tc, one in Zinaka (b) and two in Lusandwa (a).

Lusandwa (a) area had the highest trypanosomosis incidence of 28.5 % (95 % CI 23.3–34.2 %), followed by Chisulo (c) with 22.7 % (CI 18.1–28.0 %), then Zinaka (b) with 14.2 % (CI 10.5–19.0 %) and lastly Kasamanda (d) with 13.4 % (CI 10.0–17.8 %). Significant differences in disease incidence were observed between the herd in Lusandwa (a) and the herd in Zinaka (b) (*P* = 0.04) (Fig. 7b). Trypanosomosis incidence in cattle did not correlate with habitat fragmentation but showed similar patterns with mature infection rates in tsetse flies (Fig. 7a). Despite Lusandwa (a) site having a lower infection rate in tsetse flies than Chisulo (c), it had a raised trypanosomosis incidence in cattle.

#### Entomological inoculation rate

**Fig. 8:**
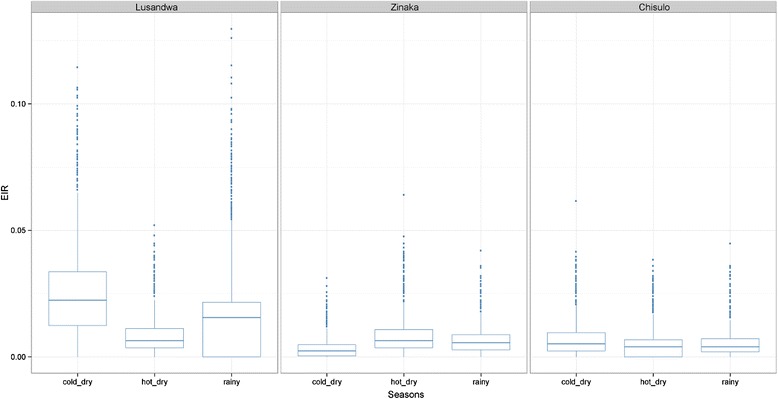
Entomological inoculation rates for the various sites and season

A total of 10,000 Monte Carlo simulations were run by bootstrapping into apparent abundance and fly infection rate to assess the EIR using study site and season as parameters. At a risk α of 5 %, it was observed that there was a significantly higher risk of trypanosomosis in Lusandwa (a) in the cold dry season than in all other seasons of other sites and in the hot dry season in Lusandwa (a) itself (Fig. 8). Overall, the risk of trypanosomosis was significantly higher in Lusandwa (a) than in the other sites.

#### Association between trypanosomosis in cattle and infection rate in tsetse populations

The OR of 0.63 (95 % CI 0.41–0.95) showed that the likelihood of cattle to be infected with trypanosomosis in Zinaka (b) reduced significantly by 37 % (*P* = 0.030) compared to Chisulo (c) cattle. In Lusandwa (a) the likelihood increased by 35 % but was not significant (OR: 1.35 CI 0.92–1.98, *P* = 0.124). The likelihood of cattle to be infected with trypanosomosis was 2.95 times higher in the rainy than in the cold dry season (OR: 2.95 (CI 2.02–4.29), *P* < 0.0001 and it increased by 48 % in the hot dry season compared to the cold dry season (OR: 1.48 (CI 0.89–2.48) (*P* = 0.130).

## Discussion

The aim of the study was to establish the status of tsetse populations inhabiting areas of varying degrees of habitat fragmentation in terms of their age distribution, abundance, proportion of female flies and infection rate. From the fragmentation indices obtained and the IAA established at study sites, it appeared that wherever host animals are available, an area with large (though few) natural patches is more favourable for *G. m. morsitans* than one with small (though numerous) natural patches as was seen at Chisulo (c) with 0.0357 IAA compared to Kasamanda (d) with 0.001 IAA. This may be a result of large natural patches providing more favourable micro-environments and breeding grounds. The same result was observed for *G. palpalis gambiensis* in Senegal [24] and Burkina Faso [13].

The following were apparent assuming that the bias of the fly round sampling method in favour of males, young flies and hungry females [25] remained constant irrespective of fragmentation (i) despite the differences in the dynamics of the IAA of *G. m. morsitans* at study sites, [increase at one site and reduction at another over the same period], it reduces as fragmentation increases (ii) the age distribution of captured *G. m. morsitans* is distorted by fragmentation in this area (iii) the proportion of female flies captured increase as fragmentation reduces (negative correlation) (Fig. 6), (iv) the proportion of old female flies increases significantly as fragmentation increases (positive correlation) (Fig. 5d), (v) the trypanosomal infection rate in *G. m. morsitans* increases significantly with fragmentation (positive correlation) (Fig. 7a). The most probable picture drawn from these results is that, whereas the population of Lusandwa (a) is a resident population breeding in all seasons, Zinaka (b) seems to be suitable for breeding during the rainy season only and Chisulo (c) site seems less suitable for breeding throughout the year. Throughout the year in Chisulo (c) and during the dry season in Zinaka (b) populations are largely made of dispersing flies. These dispersing flies are mainly old females as they are known to disperse more than younger females [26] thus distorting the age distributions to the right at these sites. Immigration may have been caused mainly by the ready availability of host animals, cattle at 8.2/km^2^.

Van den Bossche et al. [27] showed that high livestock lethality due to pathogenic strains and drug-use-induced selection pressure in favour of less pathogenic strains of *T. congolense* was responsible for the chronic form of trypanosomosis where disease transmission was mainly via a domestic cycle. As a result these less pathogenic strains became preponderant in the livestock population. Since livestock constitutes the main host for tsetse in Chisulo (c) [28] and the main trypanosomosis management regimen in eastern Zambia is drug use [29], it explains why *T. congolense* was found as the main trypanosome infecting cattle.

It must be noted, however, that identification of trypanosomes based on site of development in the fly has been shown not to be inaccurate, hence the emphasis put on global infection by authors. Otieno [30] identified *T. brucei* by inoculation in C_3_H mice of mature *T. congolense* from only mouth parts (hypopharyngeal) of infected flies.

An important factor that may play a role in higher infection rates in tsetse in fragmented areas is temperature. Burtt [31] established that there was a considerably higher infection rate of *Trypanosoma rhodesiense* (12.2 %) in flies from pupae incubated at 30 °C than in flies from non-incubated pupae (4.2 %). With flies fed on blood-positive animals transmission failures were fairly frequent in experiments with flies from non-incubated pupae. But this did not happen in flies from incubated pupae although many infections were carried out during the cooler season. Sy [32] demonstrated that flies kept at a higher temperature presented a significantly higher mature infection rate compared to flies kept at normal breeding conditions. As increased fragmentation generally increases the temperature to which tsetse are exposed, this may be the reason for the correlation between maximum daily temperature and global infection rates of tsetse flies [33].

So the higher temperatures experienced due to fragmentation at Chisulo (c) may cause a higher than normal infection rate in the locally present tsetse flies, especially during the hot season. Furthermore, a positive correlation between riverine tsetse infection rates and temperature was also observed in Burkina Faso. It resulted in a high risk of AAT at the borders between conserved and disturbed areas despite lower tsetse densities and shorter lifespan in the latter [33, 34].

Despite the tsetse population in Lusandwa (a) having a lower infection rate (7.6 %) than the Chisulo (c) population (14.5 %), trypanosomosis incidence was higher in Lusandwa (a). This was mainly due to high EIR in Lusandwa (a) that was a result of high tsetse density (about 8 times higher). For the Chisulo (c) area an incidence of trypanosomosis not significantly different from that in Lusandwa (a) (28.5 % (CI 23.3–34.2 %) and 22.7 % (CI 18.1–28.0 %), respectively) was mainly due to a high infection rate in tsetse flies despite a low tsetse density. In Kasamanda (d) a trypanosomosis incidence rate of 13.4 % (CI 10.0–17.8 %), combined with a very low density of tsetse (0.001 IAA), compared to 14.2 % incidence rate for Zinaka (b) area, must mean that the infection rate of tsetse in this highly fragmented site is probably extremely high. An association between infection rate in tsetse and cattle was observed. The low infection rate in tsetse flies at Zinaka (a) resulted in a significant reduction in the likelihood of cattle getting infected with trypanosomosis and the high tsetse infection rate at Chisulo (c) resulted in a high trypanosomosis incidence comparable to that in Lusandwa (a). The high trypanosomosis incidence at Lusandwa (a) despite low infection rate in tsetse must mean that had it not been for the high IAA of tsetse, the trypanosomosis incidence would have been lower than at Chisulo (c).

The negative correlation between the proportion of captured female flies and the degree of fragmentation was maintained even within individual seasons (Fig. 6). The underlying factor for this seems to be availability of suitable breeding grounds for female flies, which are numerous where there is good vegetation cover; hence female flies tend to shun fragmented areas. Further, observed correlations between fragmentation and biological parameters of tsetse have indicated possibilities of developing methods to structure populations based on their well-being, using integrated GIS and remote sensing technique. Some methods that could help in identifying areas where tsetse populations are most likely to persist or disappear autonomously [12], and populations likely to experience the most demographic net change [35] have been developed.

## Conclusions

Habitat fragmentation creates conditions to which tsetse populations respond physiologically and demographically thereby affecting tsetse-trypanosome interactions and hence influencing trypanosomosis risk. Despite the fly round method being inherently biased in favour of young flies, in fragmented areas old flies dominated the catches indicating that it is characteristic for populations of *G. m. morsitans* in fragmented (but habitable) areas to have high proportions of old flies. The high proportion of old flies coupled with a rise in temperature due to fragmentation, both of which increase infection rate in tsetse flies, contributes to the high risk of trypanosomosis in fragmented areas despite low tsetse density.

The correlations observed between biological parameters and the degree of fragmentation demonstrates the possibility to develop models/methods that link biological characteristics with habitat condition. Such models may be helpful in planning tsetse control interventions. Using day and night time land surface temperature and tsetse abundance data from this study, a density dependent and independent mortality model useful in identifying populations that experience varying degrees of population net changes was developed [35].

## Abbreviations

AAT: African animal trypanosomosis
EIR: Entomological inoculation rate
IAA: Index of apparent abundance
PCV: Packed cell volume
